# A tablet-based quantitative assessment of manual dexterity for detection of early psychosis

**DOI:** 10.3389/fpsyt.2023.1200864

**Published:** 2023-06-26

**Authors:** Quentin Le Boterff, Ayah Rabah, Loïc Carment, Narjes Bendjemaa, Maxime Térémetz, Anaëlle Alouit, Agnes Levy, Guillaume Tanguy, Valentine Morin, Isabelle Amado, Macarena Cuenca, Guillaume Turc, Marc A. Maier, Marie-Odile Krebs, Påvel G. Lindberg

**Affiliations:** ^1^INSERM U1266 Institut de Psychiatrie et Neurosciences de Paris, Paris, France; ^2^GHU Paris Psychiatrie & Neurosciences, Paris, France; ^3^CNRS, Integrative Neuroscience and Cognition Center, Université Paris Cité, Paris, France

**Keywords:** schizophrenia, first-episode psychosis (FEP), autism spectrum disorder (ASD), manual dexterity, behavioral marker, tablet-based assessment, Transcranial Magnetic Stimulation (TMS)

## Abstract

**Background:**

We performed a pilot study on whether tablet-based measures of manual dexterity can provide behavioral markers for detection of first-episode psychosis (FEP), and whether cortical excitability/inhibition was altered in FEP.

**Methods:**

Behavioral and neurophysiological testing was undertaken in persons diagnosed with FEP (*N* = 20), schizophrenia (SCZ, *N* = 20), autism spectrum disorder (ASD, *N* = 20), and in healthy control subjects (*N* = 20). Five tablet tasks assessed different motor and cognitive functions: Finger Recognition for effector (finger) selection and mental rotation, Rhythm Tapping for temporal control, Sequence Tapping for control/memorization of motor sequences, Multi Finger Tapping for finger individuation, and Line Tracking for visuomotor control. Discrimination of FEP (from other groups) based on tablet-based measures was compared to discrimination through clinical neurological soft signs (NSS). Cortical excitability/inhibition, and cerebellar brain inhibition were assessed with transcranial magnetic stimulation.

**Results:**

Compared to controls, FEP patients showed slower reaction times and higher errors in Finger Recognition, and more variability in Rhythm Tapping. Variability in Rhythm Tapping showed highest specificity for the identification of FEP patients compared to all other groups (FEP vs. ASD/SCZ/Controls; 75% sensitivity, 90% specificity, AUC = 0.83) compared to clinical NSS (95% sensitivity, 22% specificity, AUC = 0.49). Random Forest analysis confirmed FEP discrimination vs. other groups based on dexterity variables (100% sensitivity, 85% specificity, balanced accuracy = 92%). The FEP group had reduced short-latency intra-cortical inhibition (but similar excitability) compared to controls, SCZ, and ASD. Cerebellar inhibition showed a non-significant tendency to be weaker in FEP.

**Conclusion:**

FEP patients show a distinctive pattern of dexterity impairments and weaker cortical inhibition. Easy-to-use tablet-based measures of manual dexterity capture neurological deficits in FEP and are promising markers for detection of FEP in clinical practice.

## 1. Introduction

First-episode psychosis (FEP), i.e., the period of appearance of full-blown symptoms of schizophrenia (1) or non-primary psychosis (2), is a critical window for patient-centered care to limit chronicity (3, 4), and possibly prevent transition to schizophrenia (5). Identifying relevant biomarkers may improve early clinical detection and care of FEP, including limiting the duration of untreated psychosis and fostering personalized care by focusing on specific deficits (6). This, however, remains challenging (7). Reliable quantitative markers may assist FEP detection and follow-up (8) currently based on neuropsychological tests and subjective questionnaires.

Similarities and differences between schizophrenia and autism spectrum disorders have been previously described (9). In particular, sensorimotor impairments, which appear early in schizophrenia and are not explained by age, gender or education (10, 11), may, if assessed quantitatively, provide complementary diagnostics. Sensorimotor impairments also occur in autism spectrum disorder and are partly similar (12), partly distinct (13, 14). For instance, eye tracking (smooth pursuit) is more affected in patients with schizophrenia (15). These and other sensorimotor differences need further investigation (i) since they may reflect the neurodevelopmental load of both disorders (4), and (ii) since schizophrenia and autism spectrum disorder share genetic variants linked to motor dysfunctions in the mice model (16). Furthermore, strong sensorimotor impairments are associated with worse functional outcome in schizophrenia (17). Clinically, sensorimotor impairments are routinely assessed by neurological soft signs (NSS), which measure motor coordination and sensorimotor integration, partly reflecting cerebral dysfunction (18). NSS have been investigated as behavioral markers of schizophrenia (18–20) and of FEP (21), and have been associated with cerebellar soft signs (22–24).

In terms of underlying cerebral mechanisms, MRI investigations showed that NSS scores in patients with schizophrenia or FEP were associated with decreased gray matter volume in the motor cortex, cerebellum and other structures (25–28), and correlated negatively with cerebellar white matter structure (29). These gray/white matter alterations in schizophrenia can be accompanied by weaker cortical inhibition (30, 31) and reduced cerebellar brain inhibition (32, 33), both measured with Transcranial Magnetic Stimulation (TMS). Double pulse TMS can be used to measure GABAa inhibitory circuits of the primary motor cortex (34), and other cortical regions such as the dorsolateral prefrontal cortex (35), which is related to cognitive deficits and widely described in schizophrenia (36, 37). GABAa receptor density, implied in cortical inhibition, has also been linked to brain dysconnectivity measured with electroencephalography (EEG) in schizophrenia (38).

Here we used a tablet tool to assess sensorimotor (and related cognitive) impairments of manual control in a rapid, standardized and quantitative manner (39, 40). The first objective was to test whether patients with FEP show impaired dexterity compared to healthy control subjects, and to study whether dexterity measures allow discrimination of FEP from control participants. The second objective was to investigate whether tablet measures differentiate patients with FEP from patients with stabilized schizophrenia (SCZ) and from patients with Autism Spectrum Disorder (ASD).

Our first hypothesis, based on previous studies showing greater NSS in SCZ patients and in Ultra-High-Risk individuals for psychosis (41), was that dexterity impairments would be greater in FEP compared to controls. Second, we hypothesized that patients with FEP would show differential tablet performance compared to patients with SCZ, since NSS scores in SCZ evolve in parallel with remission of symptoms (42, 43). Third, we expected that tablet performance would also differ between patients with FEP, SCZ or ASD, since sensorimotor accuracy differs in part between the latter two (13). Hence, this would allow for discrimination of patients with FEP against patients with SCZ or ASD. Fourth, we hypothesized that manual dexterity impairments in FEP would relate to reduced cortical and cerebellar inhibition, measured with TMS (30, 33, 44).

## 2. Materials and methods

### 2.1. Participants

We used a cross-sectional design with one measurement timepoint. Participants, recruited at GHU Psychiatrie et Neurosciences hospital (Paris, France), signed an informed consent. The study was approved by the ethical review board (CPP Sud Mediterranee V nr: 2018-A01945-50, PI MO Krebs).

This pilot sample consisted of four groups, each with *N* = 20 participants, were included: a Control, FEP, SCZ and ASD group. The control, SCZ and ASD groups were age-matched, but the FEP group was younger, given that FEP is typically discovered prior to clinical diagnosis of schizophrenia (Table 1). The diagnoses were made by A.L., G.T, and V.M. before study inclusion.

**Table 1:** Demographical and clinical data for each group.

	Control	FEP	SCZ	ASD
N	20	20	20	20
Sex (M/F)	13/7	10/10	14/6	15/5
Age (years)	31 ± 9	21 ± 2*	32 ± 10	24 ± 5
Years of education	15 ± 3	12 ± 2	13 ± 3	14 ± 2
Age of first episode	NA	20 ± 2	21 ± 5	5 ± 3
STROOP (s)				
Reading	40 ± 9	48 ± 9	47 ± 8	47 ± 10
Denomination	55 ± 12	69 ± 14	71 ± 19*	68 ± 15
Interference	87 ± 21	113 ± 32	120 ± 32*	109 ± 30
PANSS				
Positive symptoms	NA	9 ± 2	10 ± 3	NA
Negative symptoms	NA	13 ± 6	15 ± 5	NA
BPRS	NA	36 ± 5	39 ± 6	38 ± 9
Medication (mg/day)	NA	182 ± 121	245 ± 267	258 ± 137 ∞

Demographical and clinical data (mean ± SD, except first two lines) for each of the four groups: Control subjects, patients with First-Episode Psychosis (FEP), with Schizophrenia (SCZ), and with Autism Spectrum Disorders (ASD). Years of education express school+higher education. Age of first psychotic episode, scores of clinical symptom scales, and medication (in mg of Chlorpromazine equivalent) were obtained for the relevant patient groups. All medicated patients received at least one atypical antipsychotic except one FEP and two SCZ patients. * denotes a significant difference from control (*=*p* < 0.05) (*t*-test). (∞) All FEP and SCZ patients and 4 ASD patients were treated with antipsychotics. The nine subjects added specifically for the decision tree analysis (6 women and 3 men) had a mean age of 31 ± 4.5 years.

The inclusion criteria were:

*For all groups*: age > 18 and < 50 years (except for FEP), be affiliated to a National Health Insurance (or equivalent) scheme.

*For patients with SCZ*: be diagnosed with schizophrenia or schizoaffective disorder under DSM-V criteria. Stable symptomatology; treated with a stable dose of antipsychotics for >1 month.

*For patients with FEP*: >18 and < 30 years old, criteria for psychosis according to Comprehensive Assessment of At Risk Mental States (CAARMS), within 2 years.

*For patients with ASD*: diagnosed using DSM-V criteria.

The exclusion criteria were:

An IQ < 70 [assessed by WAIS-IV (45)]. Standard contraindications to TMS [as in (30)]. For healthy controls: any current or previous psychiatric disease or any familial psychiatric background at the first degree.

Clinical assessments: all patients were assessed using NSS (18), Positive and Negative Syndrome Scale (PANSS) (46), Brief Psychiatric Rating Scale-24 (BPRS) (47), and the Stroop test (48). The NSS scale covers 25 items divided into four subcategories: Motor Coordination (MoCo), Sensory Integration (SI), Motor Integration (MI), and Involuntary Movement. Clinical exams were followed by manual dexterity and TMS assessments.

### 2.2. Tablet application

The tablet application tasks were developed in our lab in collaboration with Sensix.^1^ The tasks were inspired from clinical NSS procedures and previously developed measures of dexterity (49). Validity and reliability of these tablet tasks has been studied previously in healthy subjects (39). During the tablet tasks, the subject’s attention was directed to control of the fingers and use of visual/auditory task instructions and feedback. If a finger was positioned outside of the area where fingertip contacts and movements were detected, the task automatically stopped and restarted again when the fingers were correctly replaced. This procedure helped to control for drifts of attention. Furthermore, task duration was limited and pauses were inserted between tasks to reduce the risk of distraction and fatigue.

All tablet tasks were performed twice in a standardized manner (39), at least 1 h apart: first for familiarization, then for assessment. Performing all five tasks took ~20 min. The fingers must be placed and maintained on the screen during the whole task: if they moved too far, the tasks stopped. Each task is briefly described below.

**Figure 1:**
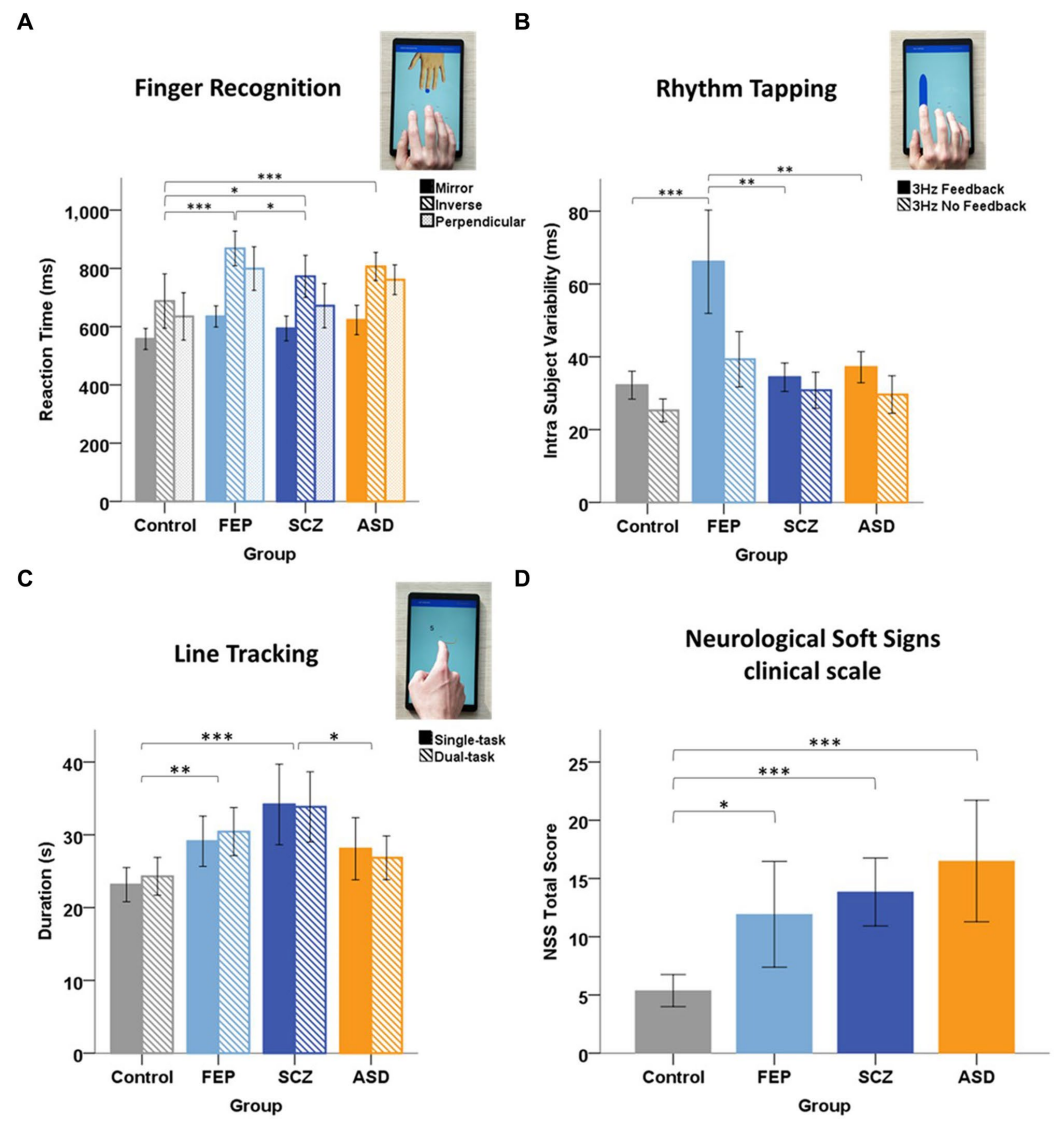
Group performance in Tablet task. **(A)** Finger Recognition task: mean reaction time in for each condition: mirror (filled bar), inverse (hatched), and perpendicular (light filled) for each group of subjects: Control, First-Episode Psychosis (FEP), Schizophrenia (SCZ), and Autism Spectrum Disorders (ASD). Error bars represent 95% CI. Horizontal brackets: significant differences with * = *p* < 0.05; ** = *p* < 0.01; *** = *p* < 0.001. *Post-hoc* tests revealed that FEP patients performed significantly slower than control and SCZ subjects, and that SCZ and ASD patients also performed significantly slower than control subjects. **(B)** Rhythm task: mean Intra Subject Variability (ISV) at 3 Hz in the *Feedback* (dark filled) and *NoFeedback* (hatched) condition, for each group. *Post-hoc* tests showed that FEP patients exhibited a larger variability (tapping irregularity) compared to control subjects in the *NoFeedback* condition and compared to all other groups in the *Feedback* condition. **(C)** Line tracking task: mean task duration in *Single-task* (dark filled) and *Dual-task* (hatched) conditions, for each group. *Post-hoc* tests revealed that SCZ patients performed significantly slower than control and ASD subjects, and that FEP also performed slower than control subjects, in both *Single* and *Dual-task* conditions. No significant differences were found between the two conditions. **(D)** Mean Total NSS score for each group. *Post-hoc* tests revealed that FEP, SCZ, and ASD patients had a significantly higher NSS Total score compared to control subjects. Total score (Kruskal-Wallis, H(3) = 25.90, *p* < 0.001) (*p* < 0.001 for SCZ and ASD) (*p* = 0.047 for FEP). But no significant differences between the three psychiatric groups were found [H(2) = 4.403, *p* = 0.111].

*Finger Recognition (FR)*: This task was designed to assess the ability for effector selection requiring mental rotation. A virtual hand appeared on the top half of the screen, opposite to the subject’s hand (Figure 1A inset). The subject had to produce a single tap, i.e., extension and flexion in a single movement, with the finger corresponding to the virtual target finger, as fast as possible and without moving other fingers (referred to as coactivations). This task consisted of three conditions: (I) *Mirror* condition: target and tapping finger were vertically aligned (Figure 1A inset), requiring no mental rotation. (II) *Inverse* condition, requiring mental rotation: the virtual hand, oriented vertically, was left/right inverted. (III) *Perpendicular* condition, requiring another type of mental rotation: the virtual hand was oriented horizontally (90° rotation). 30 trials (finger taps) were performed per condition, with 6 blocks (2 *Mirror*, 2 *Inverse*, 2 *Perpendicular*) of 5 randomized trials each.

Performance variables: reaction time, correct effector activation (% trials), and coactivation rate in non-instructed fingers (% trials).

*Rhythm Tapping (RhT)*: This task was designed to assess temporal control of finger movements. The subject had to repeatedly tap with a single finger in synchrony with an auditory cue at a given constant (1, 2 or 3 Hz) frequency (Figure 1B inset). This *Feedback* condition was immediately followed by a *NoFeedback* condition, where the subject had to continue tapping at the same frequency without auditory cue. This was repeated separately for each finger.

Performance variables: mean inter-tap-interval (ITI), intra subject variability (ISV; the standard deviation of ITI).

*Multi Finger Tapping (MFT)*: This task was designed to assess finger selection and individuation. The subject had to perform a single-finger tap or a simultaneous two-finger tap following a visual cue, as fast as possible and without moving non-target fingers. 30 single-tap trials (6 trials per finger) and 60 two-finger tap trials (6 trials per two-finger combination) were performed in randomized order.

Performance variables: reaction time, correct activations (% trials), and coactivation rate in non-instructed fingers (% trials).

*Sequence Tapping (ST)*: This task was designed to assess the ability to perform and memorize motor tapping sequences. A sequence of 5 single-finger taps was displayed on the screen and the subject was instructed to tap to each cue with the respective finger. After 10 repetitions, the subject had to repeat (5 times) the learned sequence without visual cues. Sequence_A consisted of [3-4-2-5-1] (1 = thumb, 5 = little finger) and sequence_B of [4-3-5-2-1].

Performance variables: mean number of successful taps within a trial (Success Taps Trials, range: 0–5).

*Line Tracking (LT)*: This task was designed to assess visuomotor integration and attentional capacities. The subject had to follow a curved unpredictably moving line on the screen with the index finger (Figure 1C inset). A dual-task paradigm was also implemented by adding a cognitive task: during line-tracking, shapes or single numbers (1–9) appeared briefly on the screen. The subject was instructed to ignore shapes (distractors), but to mentally subtract numbers successively from 50. Details in Supplementary methods.

Performance variables: total task duration (which depended on tracking accuracy and velocity), mean number of velocity peaks per second extracted from the entire trajectory, and number of micromovements (noise) during the task.

### 2.3. Transcranial magnetic stimulation (TMS)

#### 2.3.1. Apparatus

Subjects were seated in a chair with the right arm resting on a table. A TMS figure-of-eight Magstim coil^2^ was used with a Magstim BiStim stimulator and neuronavigation^3^ to stimulate the left hand knob of the primary motor cortex (M1). Motor Evoked Potentials (MEPs) were recorded with Ag/AgCl surface electromyography (EMG) electrodes^4^ on the First Dorsal Interosseus (FDI) and Abudctor Digiti Minimi (ADM). EMG signals were amplified with a CED 1902, sampled at 1 kHz using a CED Power1401 connected to a computer running Spike2V6.^5^ All TMS measures were obtained at rest.

The M1 hotspot was defined as the location where TMS elicited the largest FDI MEP amplitude.

#### 2.3.2. Measures of cortical excitability

*Resting motor threshold* (rMT) was defined as the percentage of maximum TMS intensity required to obtain 5/10 MEPs with amplitude >50 mV (50).

*Recruitment curve* was obtained by the slope of the mean MEP amplitude of 10 TMS pulses across each of the following intensities: 90/95/100/105/110/115 and 120% rMT.

#### 2.3.3. Measures of cortical inhibition

*Short-interval Intracortical Inhibition (SICI)* represents the inhibition of M1 given a conditioning TMS pulse 2 ms prior to a M1 test pulse (30). The conditioning pulse was at 80% rMT, the test pulse at 120% rMT. SICI was defined as the percentage reduction of the conditioned MEP relative to the unconditioned MEP.

*Cerebellar Brain Inhibition (CBI)* represents the inhibition of a M1-evoked MEP given a cerebellar conditioning pulse. The conditioning pulse, through a Magstim double-cone coil (see text footnote 2) at 50% stimulator output to minimize discomfort, was applied over the right cerebellum (5 cm lateral to occiput), 5 ms before the M1 test pulse at 120% rMT (51). CBI was quantified as percentage reduction of the conditioned relative to the unconditioned MEP.

### 2.4. Statistics

Tablet performance was analyzed with MATLAB^6^ and statistical analysis was performed with SPSS^7^ and R^8^. Normal distribution of data was assessed through Shapiro–Wilk tests. The following tablet measures were normally distributed: FR and MFT reaction times, and RhT IntraSubject Variability (ISV). TMS measures were normally distributed. Normally distributed variables were analyzed with ANOVAs with factors GROUP (Control, FEP, SCZ, and ASD), CONDITION (task-related) factors, and the CONDITION*GROUP interaction, including Bonferroni correction for post-hoc tests. RhT ISV was also analyzed for CUE and for GROUP*CUE interaction. Skewed variables were analyzed with non-parametric Scheirer–Ray–Hare tests (52, 53) with the same factors, and Dunn’s post-hoc test (corrected for multiple comparisons). The level of significance was set to *p* < 0.05. Radar plots with z-scores for each group were calculated to summarize performance results.

Correlations between tablet performance variables and age, medication, NSS outcomes, and other clinical scales were tested with Spearman’s rank-order correlation.

Receiver Operating Characteristic (ROC) curves were used to evaluate group discrimination according to area under the curve (AUC), sensitivity and specificity of three dexterity measures (which showed the most significant group differences), and of NSS. We tested FEP vs. controls (One-*vs*-One approach), then FEP vs. controls, SCZ and ASD (One-*vs*-All approach). Cut-off points were identified with the Youden Index.

A decision tree analysis based on tablet performance was computed to classify the subjects according to groups. Data from our previous study (39) of 9 healthy control subjects was added to this analysis to increase statistical power. These subjects were examined using the same protocol, but without TMS measures. Twelve performance variables with most significant group differences, three from each of four tablet tasks (FR, RhT, MFT, and LT), were used for the decision tree analysis. A complementary Random forest analysis was performed, with training and validation samples (additional information in Supplementary methods).

## 3. Results

### 3.1. Clinical and demographical data

All groups (except FEP) comprised slightly more men than women. Patients with FEP were younger; the other groups were age-matched. Age of first episode was lowest in ASD, and equivalent in FEP and SCZ. Medication was similar among patients. This and symptom scores are detailed in Table 1.

### 3.2. Group differences in tablet performance

In the *Finger Recognition task*, the ANOVA on reaction times showed a main effect of GROUP [*F*(3) = 11.995, *p* < 0.001, *η*^2^ = 0.142] and CONDITION [*F*(2) = 36.832, *p* < 0.001, *η*^2^ = 0.253], but no GROUP*CONDITION interaction [*F*(6) = 0.953, *p* = 0.458, *η*^2^ = 0.026]. The Inverse condition produced slower reaction times compared to Perpendicular (+62 ± 22 ms *p* = 0.014, Bonferroni corrected, here and in the following *post hoc* tests) and Mirror conditions (+184 ± 22 ms: *p* < 0.001). Slower reaction times were observed in Perpendicular vs. Mirror condition (*p* < 0.001). Patient groups performed significantly slower than controls: FEP (+137 ± 25 ms, *p* < 0.001), SCZ (+69 ± 25 ms, *p* = 0.040), and ASD (+114 ± 25 ms, *p* < 0.001), and FEP were slower than SCZ (+68 ± 25 ms, *p* = 0.041) (Figure 1A). Percentage of Correct trials (Supplementary Figure S1) and percentage Coactivation showed similar group differences (Supplementary Figure S2).

Taken together, these results show significant deficits in the Finger Recognition task for patients with FEP, SCZ or ASD compared to control subjects, with generally highest deficits for FEP patients.

In the *Rhythm Tapping task*, no significant group differences were found in inter-tap-interval (ITI) accuracy [Scheirer-Ray-Hare, H(3) = 7.542, *p* = 0.056]. Still, ASD patients tended to show a decreased mean ITI under auditory Feedback (40% faster than required), but a better ITI in the NoFeedback condition, resulting in the largest group difference in ITI between Feedback vs. NoFeedback conditions (Supplementary Figure S3).

**Figure 2:**
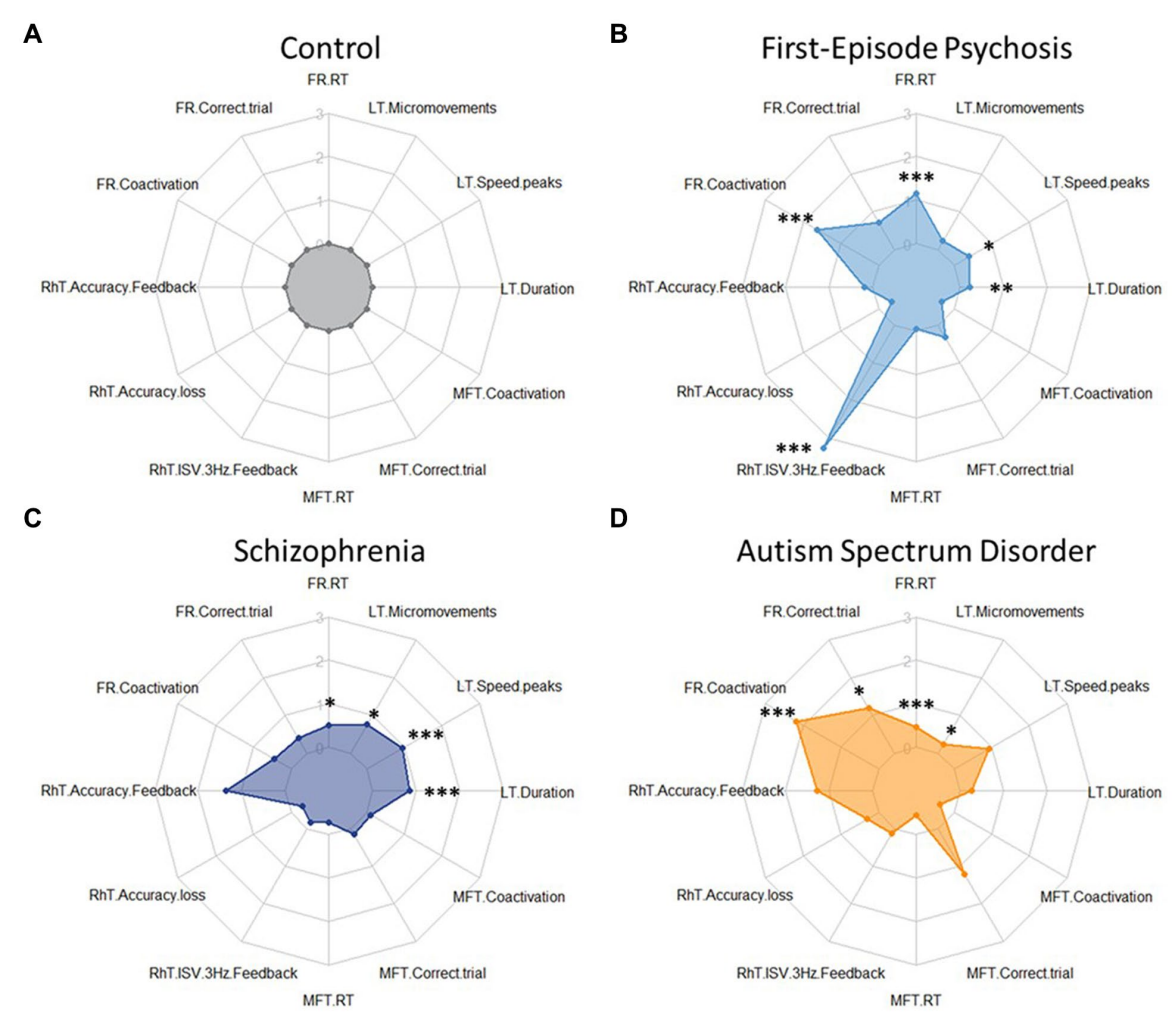
Radar plots for tablet task performance for each group Z-scores (clock-wise, starting from 12 h) for key variables differentiating group performances: Finger Recognition (FR) task: reaction time, correct trial, and coactivations in *inverse* condition; Rhythm tapping (RhT) task: inter-tap-interval *Feedback*, inter-tap-interval *Feedback*-*NoFeedback*, and Intra Subject Variability 3 Hz *Feedback*; Multi Finger Tapping (MFT) task: reaction time, correct trial, and coactivations for double taps; Line Tracking (LT) task: duration, number of velocity peaks, and number of micromovements in *single-task* condition. **(A)** Control group: performance data were normalized to 1 (and respective performance of the patient groups expressed as z-scores). Patient groups do not show generally decreased performance (i.e., uniformly increased radius of the plot), but show deficits in particular tasks and variables. **(B)** FEP patients showed higher (worse) scores in Finger Recognition (FR) reaction time and coactivations, and in Rhythm Tapping (RhT) ISV 3 Hz *Feedback*. **(C)** SCZ patients showed higher (worse) scores in Rhythm Tapping (RhT) ITI *Feedback*, ITI *Feedback-NoFeedback*, and ISV 3 Hz *Feedback*. **(D)** ASD patients showed higher (worse) scores in Finger Recognition coactivations and Multi-Finger Tapping correct trial. * show significant differences from control: * = *p* < 0.05; ** = *p* < 0.01; *** = *p* < 0.001.

The three-way ANOVA of Intra Subject Variability (ISV) showed a significant GROUP [*F*(3) = 11.485, *p* < 0.001, *η*^2^ = 0.076] (Figure 2B), CONDITION [*F*(2) = 247.145, *p* < 0.001, *η*^2^ = 0.541], and CUE effect (Feedback or NoFeedback) [*F*(1) = 182.096, *p* < 0.001, *η*^2^ = 0.303], and also a GROUP*CONDITION interaction [*F*(6) = 2.250, *p* = 0.038, *η*^2^ = 0.031]. No GROUP*CUE interaction was found [*F*(3) = 0.587, *p* = 0.624, *η*^2^ = 0.004]. Patients with FEP had a significantly higher average ISV across conditions and cues than all other groups (control *p* < 0.001; SCZ *p* < 0.001; ASD *p* = 0.006, Bonferroni corrected). At 3 Hz, patients with FEP showed a two-fold higher variability in the Feedback condition than the three other groups (Figure 1B).

No significant group differences were found in the *Multi Finger Tapping task*, nor in the *Sequence Tapping task*.

In the *Line Tracking task*, no significant differences were found between Single-and Dual-task conditions in duration, number of velocity peaks, and number of micromovements. Task duration showed significant differences for GROUP [Scheirer-Ray-Hare, *H*(3) = 22.355, *p* < 0.001] with FEP and SCZ groups being slower than controls (respectively +5 ± 3 s: *p* = 0.003 and + 10 ± 3 s: *p* < 0.001, Dunn’s test), SCZ being also significantly slower than ASD (+5 ± 3 s: *p* = 0.013) (Figure 1C). Number of velocity peaks also showed significant differences for GROUP [Scheirer-Ray-Hare, H(3) = 18.336, *p* < 0.001] with FEP and SCZ producing less smooth movement tracking than control (respectively *p* = 0.010 and *p* < 0.001, Dunn’s test). Finally, micromovements showed a significant effect of GROUP [Scheirer-Ray-Hare, H(3) = 8.893, *p* = 0.031] with ASD (Dunn’s test, *p* = 0.030) and SCZ (*p* = 0.043) exhibiting more micromovements than controls.

All significant differences presented here remained significant when comparing the FEP group with age-matched controls.

*Dexterity impairment profiles*: The performances in the four tablet tasks (in Finger Recognition, Rhythm Tapping, Multi Finger Tapping, and Line Tracking) are summarized as radar plots (Figure 2).

### 3.3. Absence of correlation between tablet performance and clinical scores

No significant correlations were found between age or medication and tablet performance variables used for patient identification (*N* = 72, all *r* < 0.261, *p* > 0.267). No consistent significant correlations were found between tablet measures and clinical scores across the 4 groups (or within the 3 patient groups for PANSS or BPRS).

### 3.4. Group differences in clinical NSS scores

The control group had significantly lower NSS total scores compared to the 3 patient groups (Figure 1D). Significantly higher scores were found in NSS subscales for the SCZ and ASD group compared to controls (Supplementary Figure S5). NSS (total or subscale) scores were not significantly different between patient groups. Patients with FEP and those with SCZ has similar PANSS scores (Total, Positive, and Negative; Supplementary Figure S7). No correlations were found between NSS scores and tablet measures.

### 3.5. Sensitivity and specificity of tablet performance variables for discriminating FEP patients from controls or other psychiatric groups

**Figure 3:**
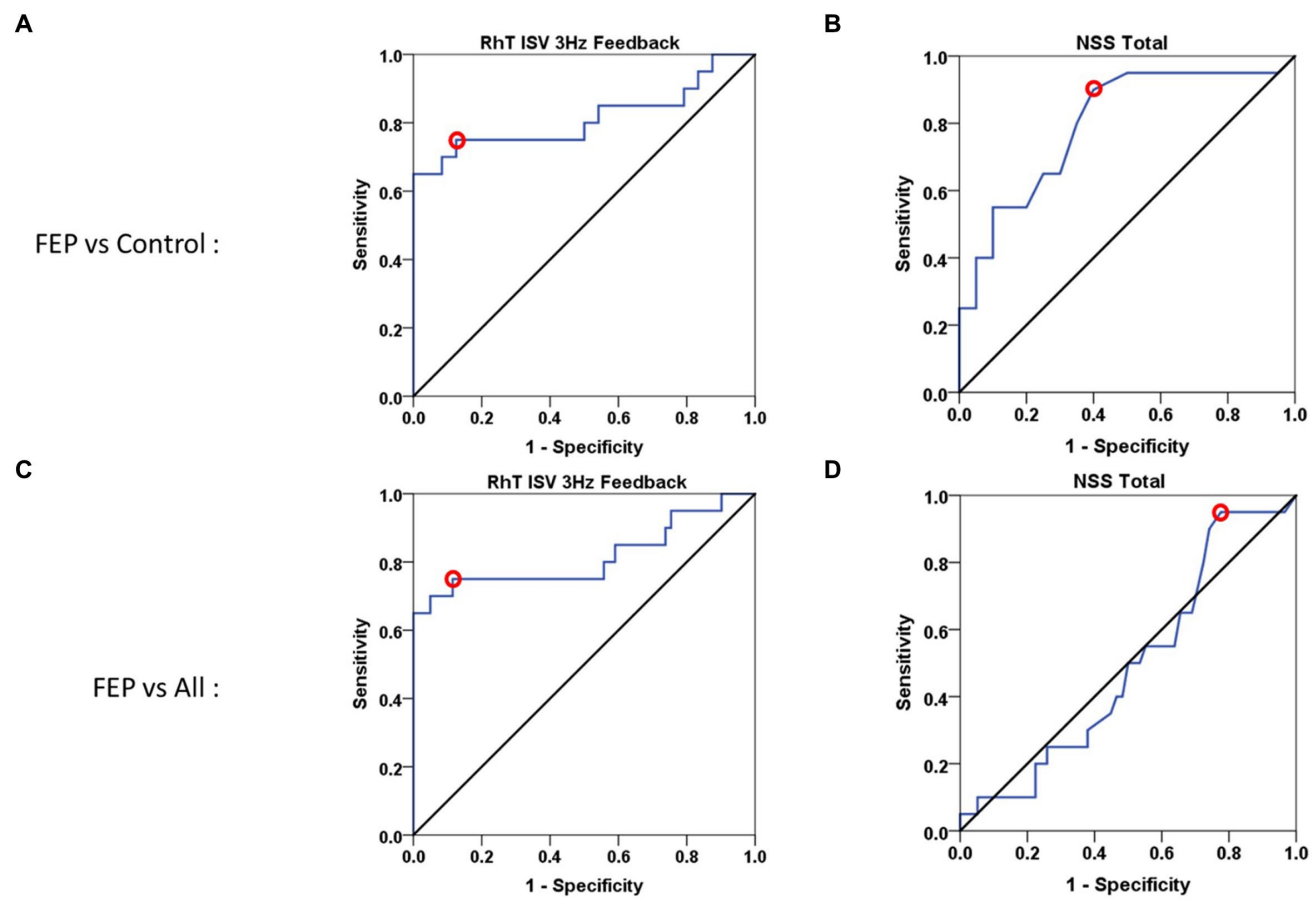
ROC curves for tablet performance and NSS score. **(A,B)** One-*vs*-One ROC curves for First-Episode Psychosis (FEP) patients vs. control subjects. **(A)** Rhythm Tapping performance (Intra Subject Variability at 3 Hz *Feedback*): sensitivity = 75%, specificity = 88.5%, Area Under the Curve = 0.812. **(B)** Clinical NSS Total score: sensitivity = 90%, specificity = 60%, Area Under the Curve = 0.797. **(C,D)** One-*vs*-All ROC curves for FEP patients vs. Control subjects and patients with Schizophrenia (SCZ) or with Autism Spectrum Disorders (ASD). **(C)** Rhythm Tapping performance (Intra Subject Variability at 3 Hz *Feedback*): sensitivity = 75%, specificity = 89.5%, Area Under the Curve = 0.815. **(D)** Clinical NSS Total score: sensitivity = 95%, specificity = 22%, Area Under the Curve = 0.492. These ROC curve results are statistically significant (*p* < 0.01 or *p* < 0.001), except for NSS total (*p* = 0.918) in the One vs. All approach [and for FR coactivations (*p* = 0.093) not shown].

Three measures, showing the most significant differences between FEP and control subjects, were used for calculating ROC curves: Reaction Time and Coactivations of the Finger Recognition task, and ISV of the Rhythm Tapping task. Tablet variables showed similar sensitivity, but better specificity and larger area under the curve than NSS scores (Table 2), whether for One-*vs*-One (Figures 3A,B) or One-*vs*-All approaches (Figures 3C,D). Similar results were found when comparing the FEP group against the SCZ group (Supplementary Figure S8).

**Table 2:** Comparative ROC curve results for three tablet performance measures and for NSS score.

Variable	One-*vs*-One ROC curve			One-*vs*-All ROC curve		
	Sensitivity	Specificity	AUC	Sensitivity	Specificity	AUC
Finger Recognition task Var: Reaction Time	93%	80%	0.81 ± 0.08	93%	70%	0.80 ± 0.05
Finger Recognition task Var: Coactivation	90%	50%	0.76 ± 0.09	90%	40%	0.65 ± 0.08
Rhythm Tapping task Var: ISV	75%	90%	0.83 ± 0.08	75%	90%	0.83 ± 0.08
NSS total score	90%	60%	0.80 ± 0.07	95%	22%	0.49 ± 0.07

One-*vs*-One analysis of FEP patients vs. Control subjects, and One-*vs*-All analysis of FEP vs. Control vs. SCZ vs. ASD. AUC, area under the curve (±SD). Variables: Reaction Time in the *Inverse* condition. Coactivation in the *Inverse* condition. ISV, Intra Subject Variability in the *3 Hz Feedback* condition.

### 3.6. Decision tree (classification) analysis

**Figure 4:**
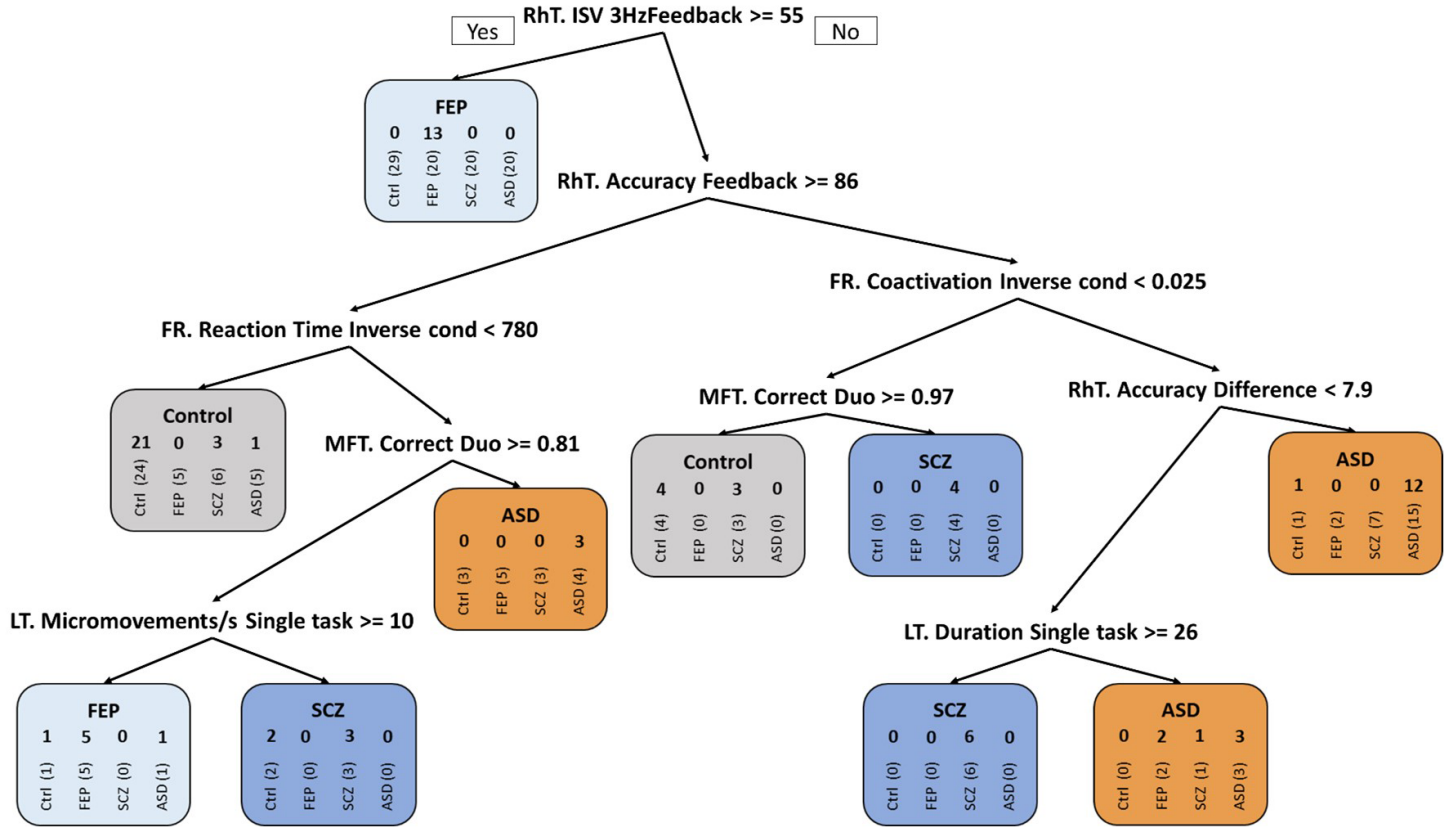
CART decision tree. Classification of the four groups using CART decision tree algorithm: Control subjects, patients with First-Episode Psychosis (FEP), with Schizophrenia (SCZ), or with Autism Spectrum Disorder (ASD). The 4 successive numbers in the rounded rectangles represent: number of control subjects, of FEP, of SCZ and of ASD patients in a particular group; e.g., if RhT ISV 3 Hz *Feedback* > = 55 ms ➔ 0 13 0 0 in the FEP group after the first branch corresponds to: 0 (among 29) control subjects (vertical writing), 13 (among 20) FEP, 0 (among 20) SCZ and 0 (among 20) ASD patients were classified as FEP patients (and so on for the following branches). Over the entire tree, false detections thus concerned 4/29 controls, 2/20 FEP, 7/20 SCZ, and 2/20 ASD patients.

The CART decision tree (Figure 4) showed a fair classification of subjects, particularly for FEP patients in the first iteration with 13/20 subjects correctly classified using the Rhythm Tapping task. Further iterations allowed the identification of 25/29 controls, 18/20 FEP patients, 18/20 ASD, but only 13/20 SCZ patients.

The complementary Random Forest analysis (Table 3) provided improved results: Good sensitivity (>92%) and specificity (>72%) for classification of control subjects vs. FEP patients. Classification of patients with SCZ or ASD showed high specificity (>90%), but low sensitivity (<38%). The model shows a 100% positive predictive value for ASD and between 50 and 70% for the other groups, while the negative predictive values scale better (between 80 and 100%), specifically for the FEP group with no false negative result. The corresponding confusion matrix from the validation sample (30% of subjects, *N* = 30) showed that the model predicted correctly 11/12 control subjects, 4/4 FEP, 3/8 SCZ, and 1/6 ASD patients (Table 3).

**Table 3:** Random Forest analysis for the four groups: control subjects, FEP patients, SCZ patients, and ASD patients.

**A**. Random Forest statistics				
	Control	FEP	SCZ	ASD
Sensitivity	92%	100%	38%	17%
Specificity	72%	85%	91%	100%
Pos. Pred. Value	69%	50%	60%	100%
Neg. Pred. Value	93%	100%	80%	83%
Balanced accuracy	82%	92%	64%	58%

Random Forest statistics over the four groups: Good classification (92% sensitivity, 72% specificity) for control subjects and for FEP patients (100% sensitivity, 85% specificity). ASD and SCZ groups showed high specificity (>90%) but low sensitivity (<38%).

Confusion Matrix of the four groups (after training on 70% of the group populations, *N* = 59): Correct classification in the validation subgroup (*N* = 30) of 11/12 control subject, 4/4 FEP patients, 3/8 SCZ patients, and 1/6 ASD patients. Thus, ASD patients were most difficult to classify/identify. See Supplementary Figure S6 for importance of the selected variables.

### 3.7. Neurophysiological (TMS) group differences and relation to tablet performance

No significant group differences were found in cortical excitation measures, i.e., for resting Motor Threshold [ANOVA, *F*(3) = 1.648, *p* = 0.186] and slope of Recruitment Curve [ANOVA, *F*(3) = 1.597, *p* = 0.198]. Detailed results in Supplementary Table S1.

**Figure 5:**
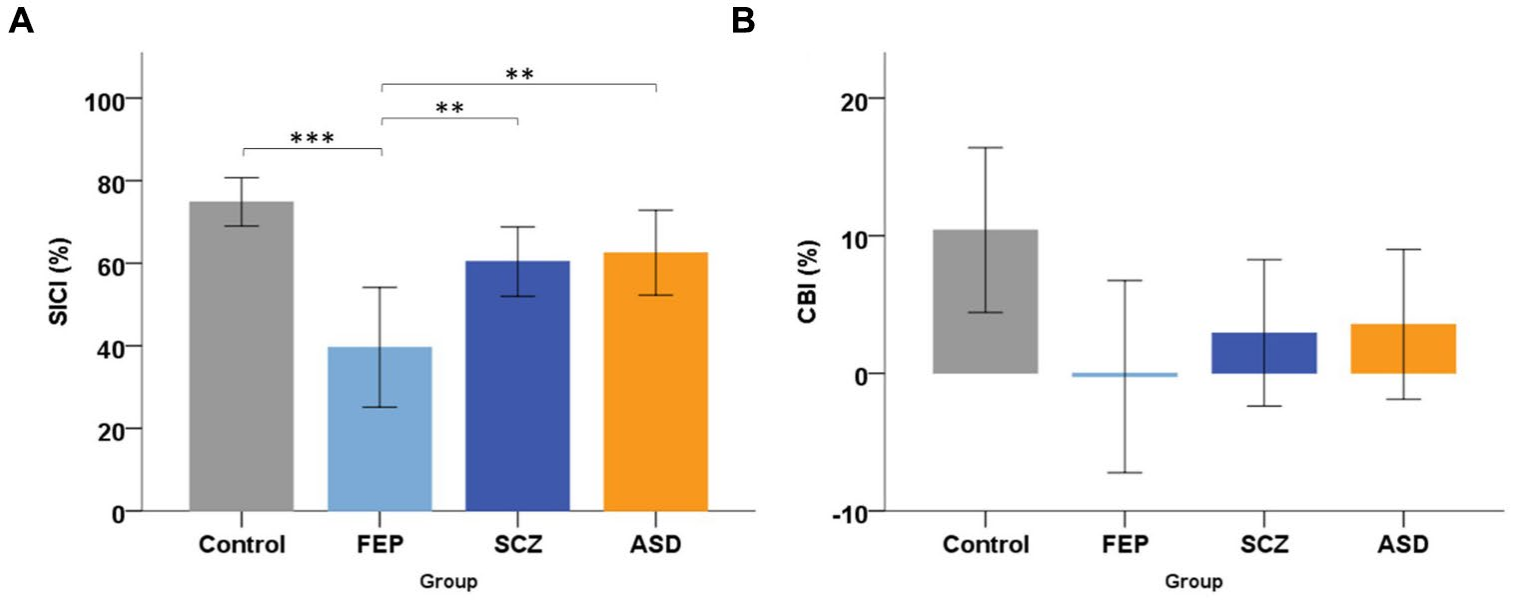
Transcranial magnetic stimulation group results. **(A)** Mean short-latency intracortical inhibition (SICI, %), for the four groups: Control subjects, patients with First-Episode Psychosis (FEP), with Schizophrenia (SCZ), or with Autism Spectrum Disorder (ASD). Error bars and horizontal brackets as in Figure 1. *Post-hoc* tests revealed that FEP patients had a significantly lower (weaker) SICI compared to Control subjects, to SCZ and ASD patients. **(B)** Mean Cerebellar Brain Inhibition (CBI, %) of M1 for the four groups. Error bars as in Figure 1. *Post-hoc* tests did not show any significant group difference in CBI. * show significant differences: * = *p* < 0.05; ** = *p* < 0.01; *** = *p* < 0.001.

Regarding motor cortex inhibition, ANOVA of SICI showed a significant effect of GROUP [*F*(3) = 8.791, *p* < 0.001]. Post-hoc showed significantly lower SICI (40%) in FEP patients (Figure 5A), compared to control subjects (75%, *p* < 0.001), to SCZ (60%, *p* = 0.025) and ASD patients (63%, *p* = 0.009). SICI was similar in SCZ (*p* = 0.271) and ASD (*p* = 0.498) compared to controls. When controlling for antipsychotic medication, the difference in SICI between FEP and SCZ remained significant (ANCOVA, *F* = 5.89, *p* = 0.021).

Cerebellar inhibition (CBI) did not show any significant effect of GROUP [ANOVA, *F*(3) = 2.433, *p* = 0.073]. Nonetheless (Figure 5B), we found weakest CBI in FEP (0% = no CBI) compared to SCZ (3%), ASD (4%) and controls (10%).

## 4. Discussion

In this study we provide novel tablet-based multi-component data on manual dexterity in patients with FEP. These data discriminate FEP from healthy controls, and from patients with SCZ and ASD. We also show a specific decrease of cortical inhibition in FEP.

### 4.1. Identification of patients with FEP through manual dexterity tasks (tablet vs. NSS)

Using direct comparison *via* ROC curves, two tablet performance variables (from the Rhythm Tapping task and Finger Recognition task) discriminated FEP patients from control subjects with similar sensitivity (~75%), but higher specificity (~90%) compared to clinical NSS scores. Better specificity was particularly clear in distinguishing FEP from Control, SCZ and ASD groups (specificity: NSS = 22% vs. dexterity>70%). The One-*vs*-All approach used here allowed distinguishing between FEP and pooled Control/SCZ/ASD groups. Good discrimination was also found between FEP and SCZ groups (Supplementary Figure S8). One-*vs*-all comparison was undertaken to test the tablet in an ecological manner, with the rationale of detecting FEP against a population with large variability, i.e., approaching that of the general population, in order to use the tablet as a screening instrument for detection of at-risk population.

Decision tree and Random forest analysis, that use (in contrast to ROC curves) multiple tablet performance measures, were applied to attempt distinguishing subjects of each group. Detection of FEP patients through these methods corroborated our ROC results: according to the positive and negative prediction values and balanced accuracy, patients with FEP were distinguished with least error, followed by Control subjects and patients with SCZ. Classification of patients with ASD was most difficult. Thus Decision tree and Random Forest analysis allow for partial discrimination between all four groups. Although promising, the model requires larger samples to be improved. Together, quantitative tablet-derived measures of manual dexterity provide better behavioral diagnostic markers of FEP than clinical NSS.

### 4.2. Comparison to other markers

Rapid, easy-to-use, quantitative and non-subjective tools remain a key challenge in psychiatry (54). Several approaches have been investigated to identify SCZ and/or FEP by symptom/neuropsychological criteria, by other behavioral abnormalities (e.g., gaze), by cognitive deficits (attention, language), or by biological markers (brain imaging, genotype) (55). It seems therefore worthwhile to compare the detection accuracy of the tablet markers against other markers.

First, symptom criteria: Clinical scales can detect FEP with ~77% sensitivity and ~ 70% specificity among new referrals to mental health services (56), though patients with mild symptoms remain typically undetected (57). Second, other behavioral markers: gaze abnormalities, e.g., antisaccades or number of fixations (58–63), show comparable FEP detection accuracy (~77% sensitivity, ~81% specificity). Third, cognitive abilities: abnormal language production detects FEP patients with sensitivity and specificity of about 70–80% (64, 65). Our dexterity measures thus achieved similar or better sensitivity and specificity than most other symptomatological/behavioral/cognitive approaches. However, none of these markers achieves sufficient detection accuracy on its own, but they may be useful in early screening, which would need complementary confirmation through more costly, but typically more accurate biological markers, whether molecular (66) or brain imaging (55, 67–69). More generally, a multimodal approach to FEP detection seems to be warranted.

### 4.3. Sensorimotor aspects of group differences in manual dexterity (tablet tasks)

As expected, patients with FEP or SCZ showed deficits in tablet performance, in line with previous studies on sensorimotor deficits assessed by NSS in SCZ (18, 43, 70, 71) and in Ultra-High-Risk individuals (41). We further confirmed (Figure 2) differential group deficits in manual dexterity in patients with ASD (13). More specifically, weaker performance in Finger Recognition is consistent with slower and less accurate mental rotation in SCZ (72, 73) and FEP (74), but not in ASD (75, 76). In Rhythm Tapping, only FEP patients were impaired, in contrast to deficient temporal perception shown in SCZ and ASD (77, 78), a discrepancy likely due to task differences. Sensorimotor accuracy in Line Tracking was affected in SCZ and FEP groups, consistent with previous findings (30, 79), whereas ASD patients showed good tracking performance, unlike previously reported using grip force control measures (13). Expected group differences in divided attention, operationalized by the Line Tracking Dual-task condition, were not found, likely due to sub-optimal tablet task conditions.

### 4.4. TMS-related neurophysiological markers

Although there were no significant group differences in Cerebellar Brain Inhibition, the non-significant trend for reduced CBI in FEP vs. controls is in line with previous reports in SCZ (33).

However, significantly reduced SICI in FEP, and trends to reduced SICI in ASD and SCZ, were consistent with previous results in FEP (44), ASD (80–82), and SCZ (30, 83, 84). Age disparity (younger FEP subjects) is not likely to explain this SICI group difference since SICI was shown to not relate to age (85). We found no evidence for altered cortical excitability in patient groups, in line with our previous report (30), but in contrast to other studies (86, 87). Together these results suggest that reduced SICI is a stronger marker for FEP, a neurodevelopmentally volatile phase (5), than for SCZ or ASD. This needs confirmation in larger studies and might be expanded to Ultra-High-Risk patients.

### 4.5. Limitations

Here we discuss potential issues that may have affected our data and biased the results. This was a pilot study and our results need to be confirmed in larger samples. Expansion to ultra-high risk (UHR) subjects could provide valuable insights to sensorimotor signatures present early in the development of psychosis. Regarding the measures used, the (18) NSS scale, associated with structural motor cortex alterations (70), is not the only available NSS scale. We do not know whether other NSS scales would have given similar results. Subclinical attentional deficits may have affected our dexterity measures (30). We attempted (without success) to quantify divided attention through the dual-task condition in the line-tracking task. Age difference across groups, unavoidable in comparisons of FEP against the other groups, may have contaminated results. However, we did find a similar difference in tablet performance in FEP when comparing to an age-matched sub-sample. Another potential methodological bias was that we added 9 control subjects in the classification analysis. However, FEP remained the best classified group even with 20 subjects in each group. Medication did not seem to confound our results since including medication in the ANCOVA analysis gave similar results. We did not have information on whether patients had received psychotherapy, which can improve symptoms and functioning of psychotic patients (88, 89). Regarding the interpretation of results, one could also argue that we did not distinguish FEP from SCZ and ASD, but only discriminated between acute psychotic disorder (FEP) and chronic psychotic disorder [as in SCZ and ASD; (4)]. However, patients with FEP and those with SCZ had similar PANSS scores (see Supplementary Figure S7), suggesting that our measures of dexterity capture sensorimotor aspects particular to FEP and not explained by severity of psychotic symptoms. Finally, in our TMS measurements of CBI we used rather low and constant stimulation intensity (to avoid discomfort) and CBI may have been stronger if using higher stimulation intensities (51).

### 4.6. Conclusion

Patients with first-episode psychosis showed impairments in manual dexterity compared to control subjects when assessed by a new tablet application. Differential manual deficits were also present in patients with schizophrenia or autism spectrum disorder. Tablet-based measures, used as behavioral markers, allowed identification of first-episode psychosis patients vs. control subjects, and differentiated FEP from patients with schizophrenia and patients with autism spectrum disorder with good sensitivity and specificity, and more accurately than detection using neurological soft signs. Neurophysiological alterations were also present with a strongly reduced cortical inhibition in patients with first-episode psychosis. The behavioral results suggest that tablet-based measures of manual dexterity may serve as promising complementary markers for the detection of first-episode psychosis. Replication in a larger cohort is currently under investigation.

## Data availability statement

The raw data supporting the conclusions of this article will be made available by the authors, without undue reservation.

## Ethics statement

The studies involving human participants were reviewed and approved by CPP Sud Mediterranee V nr: 2018-A01945-50, PI MO Krebs. The patients/participants provided their written informed consent to participate in this study.

## Author contributions

MM, M-OK, and PL designed the study. QL conducted the analysis and wrote first draft. AR, LC, MT, and AA collected eNSS and TMS data. LC designed the tablet application and data extraction scripts. NB performed clinical assessments. AL, GTa, VM, IA, and MC recruited patients and obtained patient consent. GTu supervised decision tree analysis. PL supervised data collection and analysis. MM and PL revised the drafts. All authors contributed to revision of the final draft and approved it.

## Funding

AR and LC were financed by Fondation Pierre Deniker (Fondation de l’Avenir, FS-MAT-17), and QL was financed by the reference ANR-18-RHUS-0014 (“Project PsyCARE”).

## Conflict of interest

MT and PL own shares in Dextrain company (www.dextrain.com) which develops and commercializes solutions for measurement and rehabilitation of manual dexterity. MT, MM, and PL have a patent on the DexTrain manipulandum (WO2020070305A1) and method for evaluating manual dexterity (WO2016184935A2).

The remaining authors declare that the research was conducted in the absence of any commercial or financial relationships that could be construed as a potential conflict of interest.

## Publisher’s note

All claims expressed in this article are solely those of the authors and do not necessarily represent those of their affiliated organizations, or those of the publisher, the editors and the reviewers. Any product that may be evaluated in this article, or claim that may be made by its manufacturer, is not guaranteed or endorsed by the publisher.

## Footnotes

^1^ www.sensix.fr

^2^ www.magstim.com

^3^ https://www.ant-neuro.com/products/visor2

^4^ www.adinstruments.com

^5^ www.ced.co.uk

^6^ www.mathworks.com

^7^ www.IBM.com

^8^ https://www.r-project.org/

## Glossary

ASD: Autism Spectrum Disorder
BPRS: Brief Psychiatric Rating Scale
CAARMS: Comprehensive Assessment of At-Risk Mental States
CART: Classification and Regression Trees
CBI: Cerebellar Brain Inhibition
FEP: First-Episode Psychosis
FR: Finger Recognition
ISV: Intra Subject Variability
LT: Line Tracking
M1: Primary motor cortex
MEP: Motor Evoked Potential
MFT: Multi Finger Tapping
NSS: Neurological Soft Signs
PANSS: Positive and Negative Syndrome Scale
RC: Recruitment Curve
RhT: Rhythm Tapping
rMT: resting Motor Threshold
ROC: Receiver Operating Characteristic
RT: Reaction Time
SCZ: Schizophrenia
SICI: Short-latency IntraCortical Inhibition
ST: Sequence Tapping
TMS: Transcranial Magnetic Stimulation
