# Supplementary material for: A tablet-based quantitative assessment of manual dexterity for detection of early psychosis
Source: Front Psychiatry. 2023 Jun 26;14:1200864. doi: 10.3389/fpsyt.2023.1200864 (PMC10330763; doi:10.3389/fpsyt.2023.1200864)
Supplement: Supplementary file 1 [file Data_Sheet_1.PDF]

Supplementary Material

A tablet-based quantitative assessment of manual dexterity for detection of early psychosis

Quentin Le Boterff [1*], Ayah Rabah [1], Loïc Carment [1], Narjes Bendjemaa [1,3], Maxime Térémetz [1], Anaëlle Alouit [1], Agnes Levy [3], Guillaume Tanguy [3], Valentine Morin [3], Isabelle Amado [3], Macarena Cuenca [3], Guillaume Turc [1, 3], Marc A. Maier [2], Marie-Odile Krebs [1, 3] & Påvel G. Lindberg [1]

[1] INSERM U1266 Institut de Psychiatrie et Neurosciences de Paris, 75014 Paris, France

[2] Université Paris Cité, CNRS, Integrative Neuroscience and Cognition Center, F-75006 Paris, France

[3] GHU Paris Psychiatrie & Neurosciences, 75014 Paris, France

*** Correspondence:**
Quentin Le Boterff
qu.leboterff@gmail.com

Supplementary methods

Tablet application (set-up):

At the beginning of each task (except for Line Tracking), a blue rectangle was displayed on the lower half of the tablet screen, indicating where the fingers had to be positioned. The tablet detects and records for each timeframe the position (X, Y pixels) of each finger pad on the screen. A task began, when all five fingers were detected in the lower half and were in stable contact with the screen. Standardized oral instructions were given before each task.

Line Tracking:

During the *simple-task* condition, a snake-like trajectory appeared on the screen and progressed according to the subject's index finger velocity, always displaying 1200 pixels of the upcoming trajectory. When the finger was too far from the line (>100 pixels), the trajectory stopped until the

index finger was sufficiently close and then started moving again. The subject was instructed to perform line-tracking as fast and as accurately as possible. The *dual-task* condition added a cognitive task: during line-tracking, shapes (Triangle, Circle, Star, Square) or single numbers (1 to 9) appeared briefly on the screen. The subject was instructed to ignore the shapes, which were distractors, but to attend to the numbers, in order to mentally subtract them successively from 50 while line-tracking.

<u>*Decision tree analysis: inclusion of other data, and treatment of missing data*</u>

Decision tree analysis was previously used to classify patients with psychiatric disorders (e.g. for differential diagnostics as in Léger et al., 2022; Dong et al., 2022). The Classification and Regression Tree (CART) procedure in R was used for computing decision trees. Ten subjects in the node was the minimal value in order to have a split. Our previous study (Rabah et al., 2022), from which data of 9 additional healthy controls were included, used identical tablet tasks (and respected similar inclusion criteria). In total, 78 missing data (for 39 subjects across all variables) were imputed with the median values of the respective group, representing 7.3% of the total variables, and the correlations between the data (Jadhav et al., 2019) were low, with max r<0.50 and only one p-value<0.001.

<u>*Random forest analysis in R*</u>

This consisted of bootstrapping the data to create multiple decision trees (500), and then apply featured randomness (i.e. picking random features from each tree to construct the final data). Random forest used the data of 70% of the group populations (N=59) as training set. The classification was then validated on the remaining N=30 subjects. Bootstrapping and featured randomness are designed to increase the variation between the trees and thus limit overfitting (Breiman, 2001).

Supplementary Table 1: TMS values (mean±SD) representing cortical excitation for the four groups (Control subjects, FEP patients, SCZ patients and ASD patients)

	Control	FEP	SCZ	ASD
rMT	49.1±6.8%	52.3±8.7%)	52.6±6.6%	54.8±9.3%
MEP amplitude	140±124mV	67±72mV	103±98mV	103±97mV
Slope of RC	0.34±0.22	0.19±0.18	0.26±0.20	0.27±0.21

Excitation measures show the following gradient trends:

rMT: Control < FEP < SCZ < ASD

MEP amplitude: Control > SCZ ~ ASD > FEP

Slope: Control > ASD > SCZ > FEP

Supplementary Figure S1. Group performance in the three conditions of the Finger Recognition task. Performance variable: Coactivation.

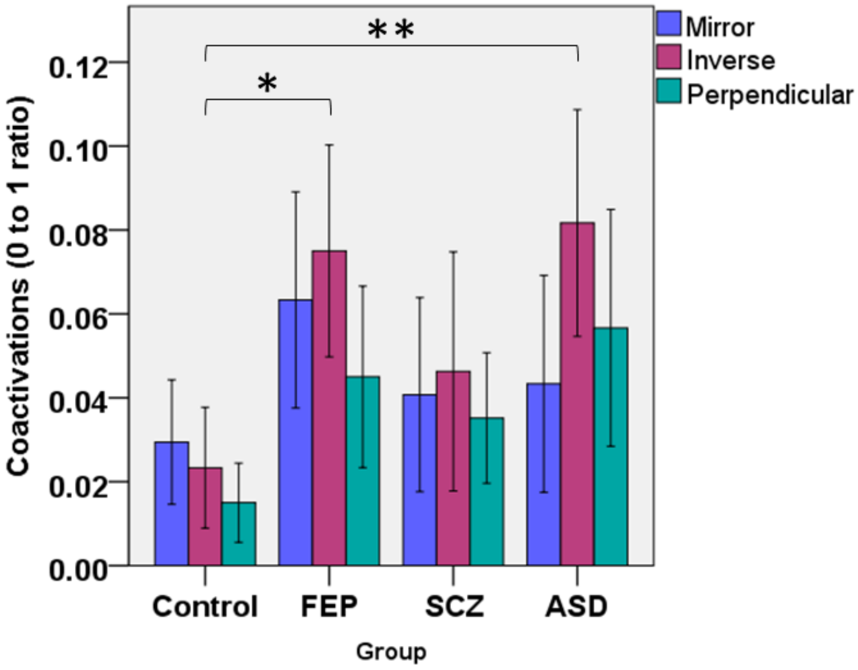

Coactivations, i.e. unwanted extra finger taps, were more frequent for FEP (8%, Dunn's test, $p<0.001$) and ASD (8%, $p<0.001$) compared to controls (2%). And FEP also showed more coactivations than SCZ (4%, $p=0.041$). Coactivations were significantly different in GROUP (Scheirer-Ray-Hare, $H(3)=23.299$, $p<0.001$), but no effect was found for CONDITION (Scheirer-Ray-Hare, $H(2)=4.500$, $p=0.105$) nor the interaction GROUPxCONDITION (Scheirer-Ray-Hare, $H(6)=6.791$, $p=0.341$).

Supplementary Figure S2. Group performance in the three conditions of the Finger Recognition task. Performance variable: percentage of Correct trials.

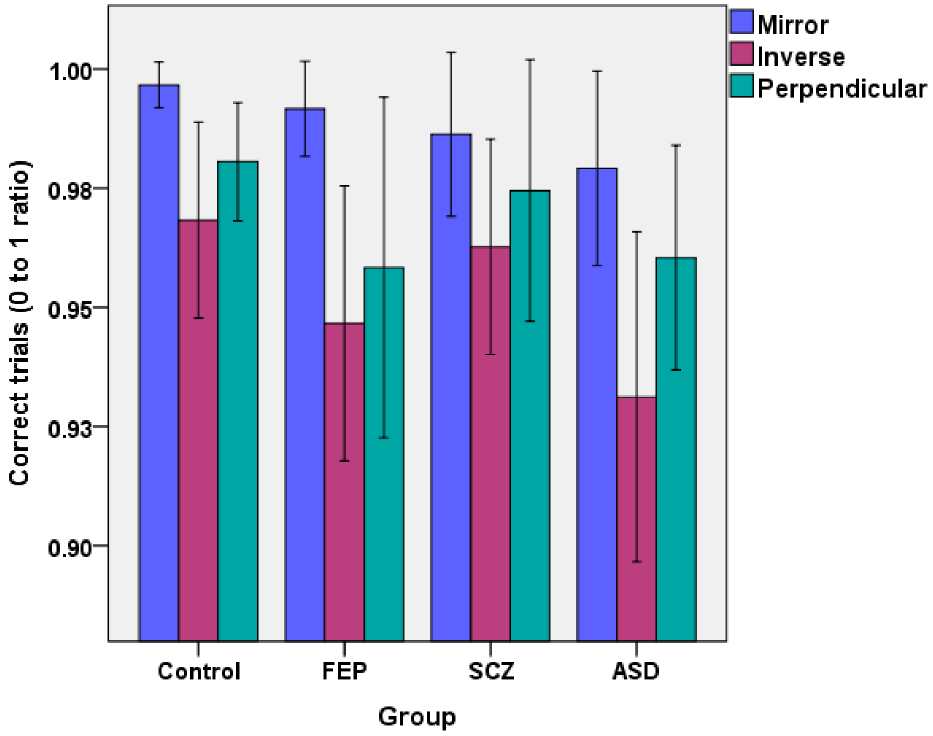

The percentage of Correct trials was significantly different across both GROUP (Scheirer-Ray-Hare, $H(3)=10.165$, $p=0.017$) and CONDITION (Scheirer-Ray-Hare, $H(2)=24.533$, $p<0.001$), but not GROUPxCONDITION (Scheirer-Ray-Hare, $H(6)=0.853$, $p=0.991$). Again, *Inverse* condition showed worse results compared to both *Perpendicular* ($p=0.041$, Dunn's test) and *Mirror* conditions ($p<0.001$, Dunn's test), while *Perpendicular* was linked to worse results compared to Mirror condition ($p=0.006$). After correction, only ASD remained worse in FR than control ($p=0.014$, Dunn's test).

Supplementary Fig S3. Group performance in ITI Accuracy of the Rhythm Tapping task.

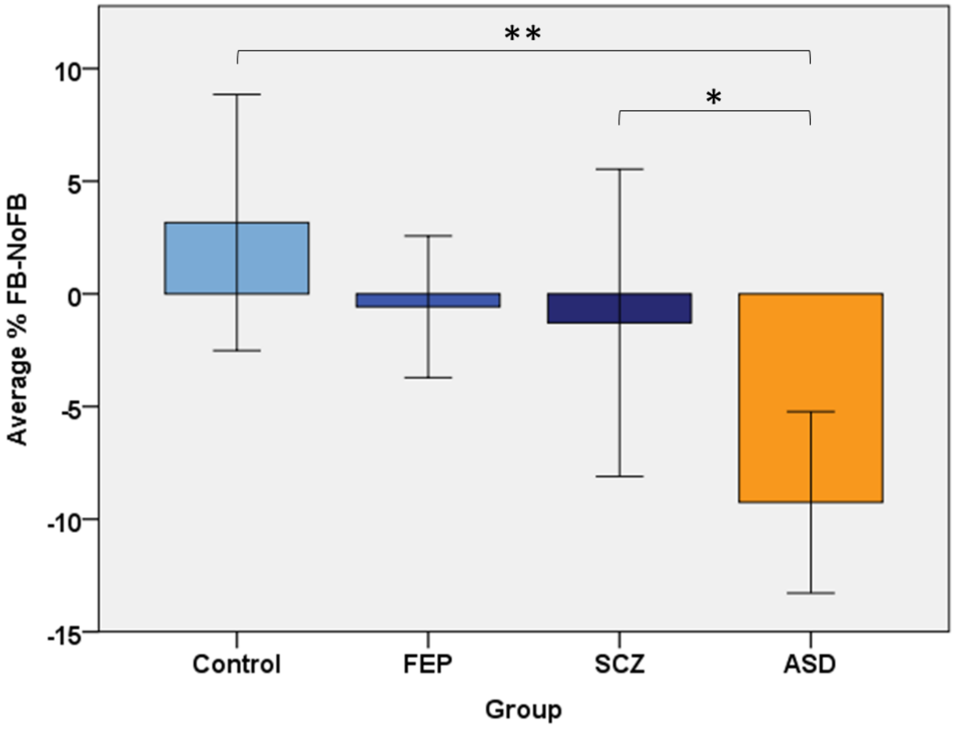

Mean difference of accuracy (%) of Inter Tap Interval between the Feedback (FB) and No Feedback (NoFB) conditions, for the four groups: Control subjects, patients with First-Episode Psychosis (FEP), with Schizophrenia (SCZ), or with Autism Spectrum Disorder (ASD). Positive values indicate a better accuracy in FB than in NoFB. Error bars represent 95% CI. Horizontal brackets: significant differences with * = $p<0.05$; ** = $p<0.01$; *** = $p<0.001$. Post-hoc tests revealed that ASD patients were significantly more precise in the NoFB than in the FB condition, compared to Control subjects and to SCZ patients.

Supplementary Fig S4. Group performance in the two conditions of the Line Tracking task.

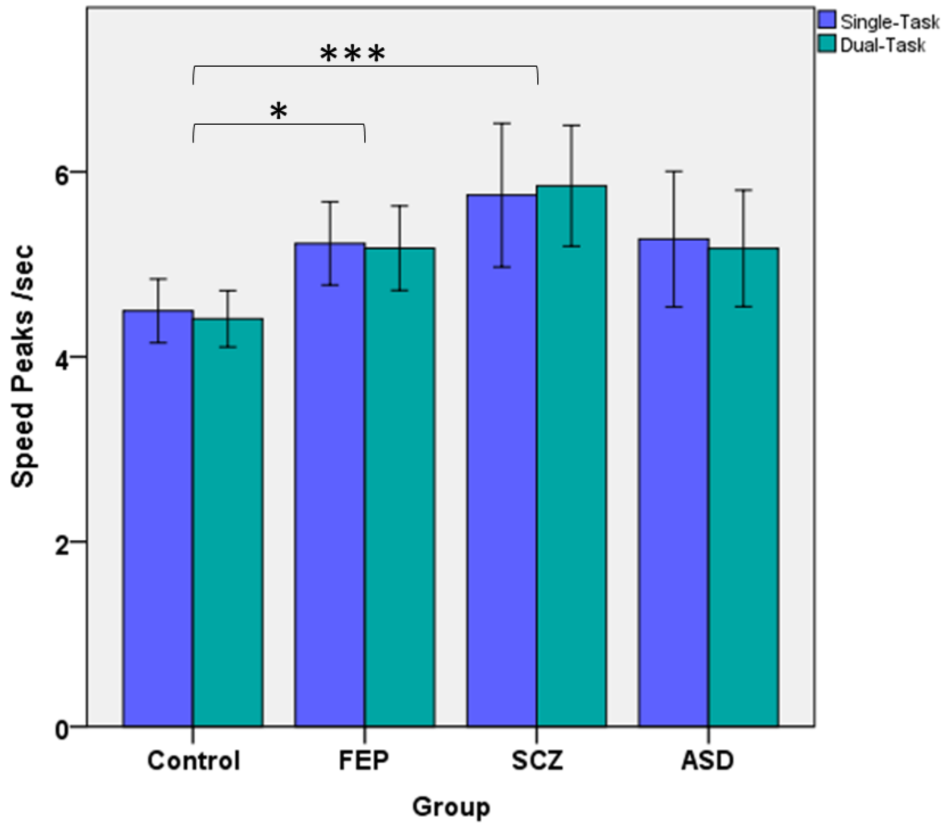

FEP and SCZ patients showed more saccadic movements (measured by number of velocity peaks /seconds) than Control (Scheirer-Ray-Hare, $H(3)=18.336$, $p<0.001$ / respectively $p=0.010$ and $p<0.001$, Dunn's test).

Supplementary Figure S5. Group differences in NSS clinical subscales.

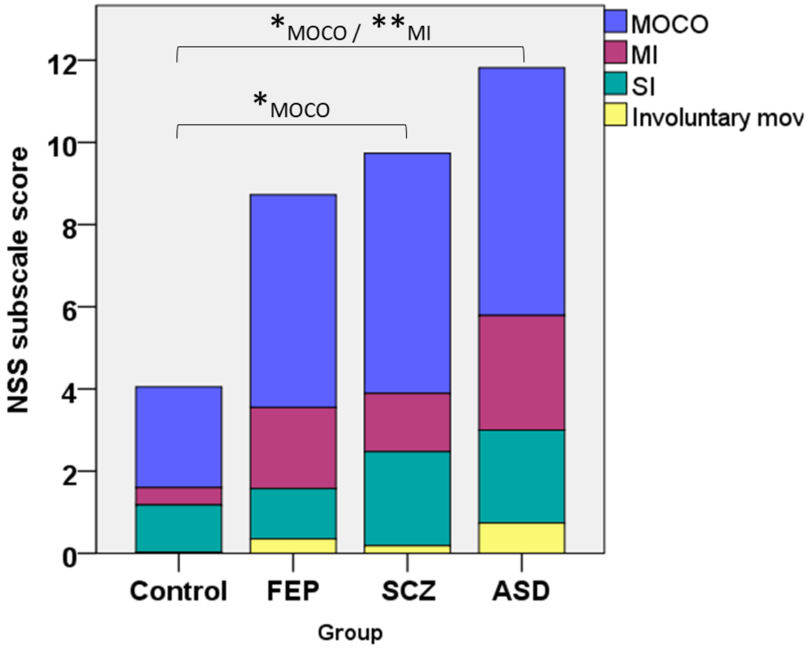

Significant differences were found at the level of NSS subscales for the SCZ and ASD group, (MoCo (H(3)=13.088, p=0.004) and MI (H(3)=15.983, p=0.001), but not for the FEP group (MoCo: p=0.124 / MI: p=0.057). ASD patients showed significantly higher deficits than Control subjects in Motor Coordination and Motor Integration subscales. SCZ patients showed a significantly higher deficit than Control subjects in Motor Coordination (MOCO).

No further significant differences were found in other NSS scores (SI: p=0.118, Involuntary Movements: p=0.269). However, all patient groups showed Involuntary Movements, but control subjects did not.

Supplementary Figure S6. Variable importance in the Random Forest analysis.

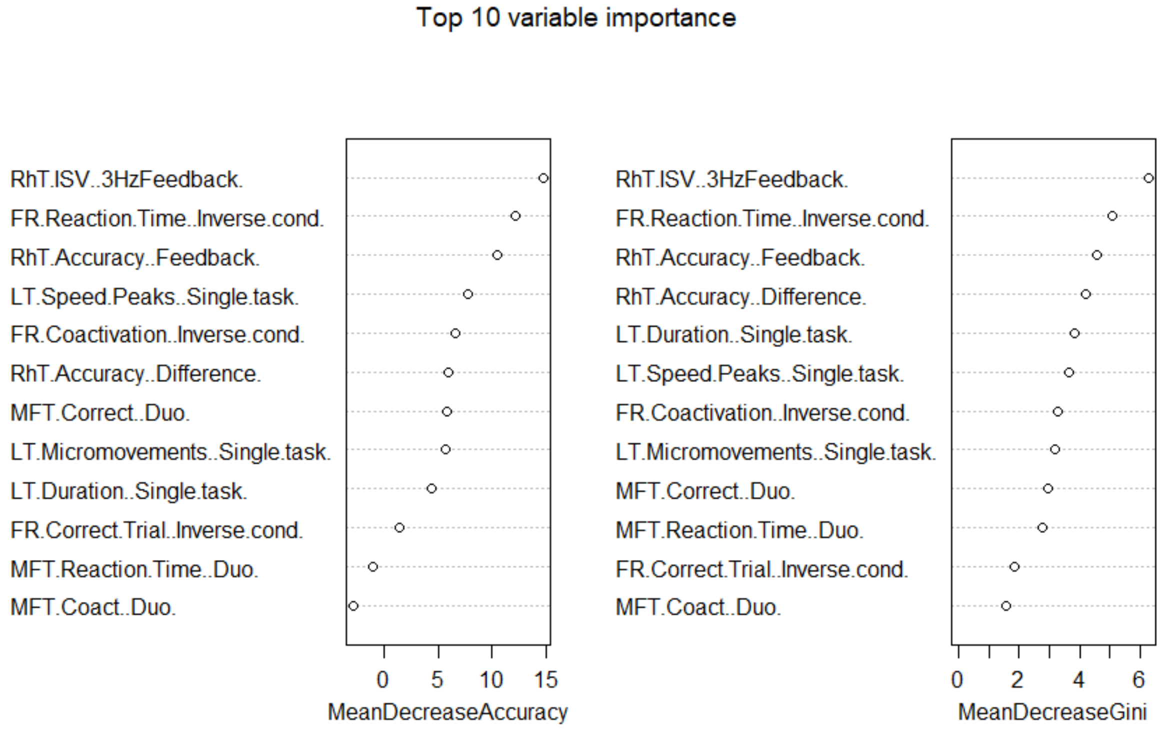

The left panel classes the variables by the accuracy they convey to the model. The right panel classes the variables by the purity they pass to the model. Note: qualitative similar results are expressed in the radar plots of Fig. 2. RhT: Rhythm Tapping task; FR: Finger Recognition task; LT: Line Tracking task; MFT: Multi Finger Tapping task.

Supplementary Figure S7. Distribution of PANSS scores for FEP and SCZ individuals.

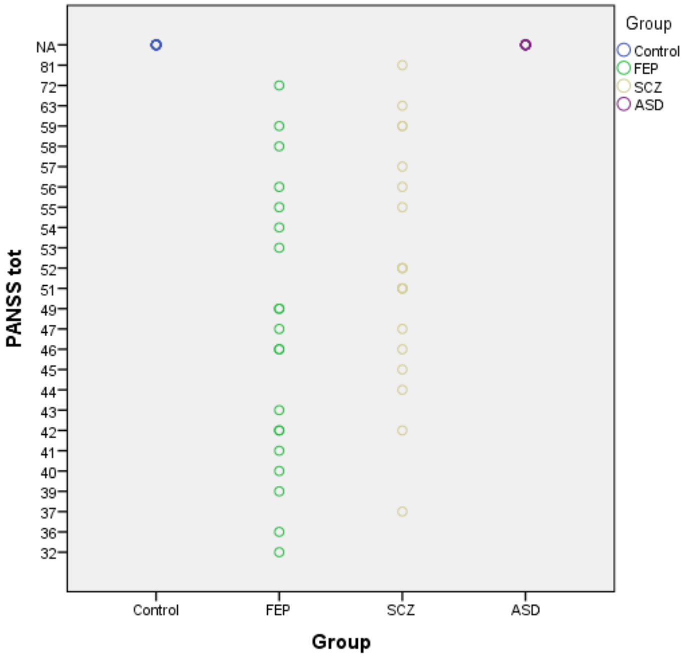

Distribution of the PANSS total score in FEP and SCZ subjects (ASD and Control subjects did not pass this scale). FEP and SCZ scores are similarly distributed.

Supplementary Figure S8. ROC Curves FEP vs SCZ only.

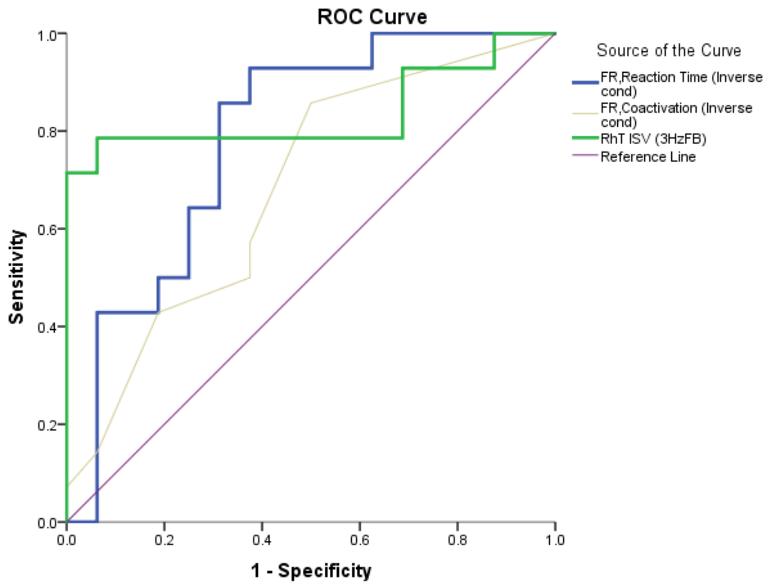

Area Under the Curve

Test Result Variable(s)	Area
FR,Reaction Time (Inverse cond)	.786
FR,Coactivation (Inverse cond)	.683
RhT ISV (3HzFB)	.835

On the left: ROC curves for FR Reaction Time, FR Coactivation, and RhT ISV 3Hz FB in identification of FEP against SCZ. On the right: AUC for these measures.

References:

Breiman, L., 2001. Random Forests. Machine Learning 45, 5–32. https://doi.org/10.1023/A:1010933404324

Dong, W., He, Y., Wang, J., Shi, C., Niu, Q., Yu, H., Ji, J., Yu, X., 2022. Differential diagnosis of schizophrenia using decision tree analysis based on cognitive testing. The European Journal of Psychiatry 36, 246–251. https://doi.org/10.1016/j.ejpsy.2022.05.003

Jadhav A, Pramod D, Ramanathan K. Comparison of Performance of Data Imputation Methods for Numeric Dataset. *Applied Artificial Intelligence*. 2019 ;33(10):913-933. doi:10.1080/08839514.2019.1637138
